# Systematic review and meta-analysis of randomised trials and cohort studies of mycophenolate mofetil in lupus nephritis

**DOI:** 10.1186/ar2093

**Published:** 2006-12-12

**Authors:** R Andrew Moore, Sheena Derry

**Affiliations:** 1 Pain Research and Nuffield Department of Anaesthetics, University of Oxford, The Churchill, Headington, Oxford OX3 7LJ, UK

## Abstract

Mycophenolate mofetil (MMF) is an immunosuppressant drug being used for induction and maintenance of remission of lupus nephritis in systemic lupus erythematosus. Evidence about its use was sought from full publications and abstracts of randomised trials and cohort studies by using a variety of search strategies. Efficacy and adverse event outcomes were sought. Five randomised trials enrolled patients with World Health Organization (WHO) class III, IV, or V (mostly IV) lupus nephritis, predominantly comparing MMF (1 to 3 g daily) with cyclophosphamide and steroid. Complete response and complete or partial response was significantly more frequent with MMF than with cyclophosphamide, with numbers needed to treat of 8 (95% confidence interval 4.3 to 60) to induce one additional complete or partial response, with wide confidence intervals. Death was reported less frequently with MMF (0.7%, 1 death in 152 patients) than with cyclophosphamide (7.8%, 12 deaths in 154 patients), with a number needed to treat to prevent (NNTp) one death of 14 (8 to 48). Hospital admission was also lower with MMF (1.7% versus 15%; NNTp 7.4 [4.8 to 16]). Serious infections, leucopaenia, amenorrhoea, and hair loss were all significantly less frequent with MMF than with cyclophosphamide, but diarrhoea was significantly more common with MMF. Ten of 18 cohort studies enrolled only patients with lupus nephritis (author-defined or WHO class III to V). Seven of these 10 reported that complete or partial response with MMF (mostly 1 or 2 g daily) with steroid occurred in 121/151 (80%) and that treatment failure or no response occurred in 30/151 (20%). Adverse events were generally similar in cohort studies with and without only patients with lupus nephritis. In all 18 cohorts, gastrointestinal adverse events (diarrhoea, nausea, vomiting) occurred in 30%, infection in 23%, and serious infection in 4.3%. Adverse event discontinuations occurred in 14% and lack of efficacy occurred in 10%. There was a single death with MMF, a mortality rate over the course of 1 year of approximately 0.2%. The results form a basis on which to plan future studies and provide a guide for the use of MMF in lupus nephritis until results of larger studies are available. At least one such study is under way.

## Introduction

The prevalence of systemic lupus erythematosus (SLE) varies with age, gender, and ethnicity, and the highest rates occur in young adult women, particularly of Afro-Caribbean origin, who are in peak childbearing years [1-4]. Nephritis complicates SLE in a significant minority of patients and is associated with renal failure and increased mortality. The tendency for people of Afro-Caribbean origin to have a worse prognosis may be due, at least in part, to poor socio-economic status [5]. In the 1950s, patients with class IV nephritis rarely lived longer than 5 years, whereas now more than 80% survive with good renal function for more than 10 years [6].

The World Health Organization (WHO) classification for lupus nephritis is based on the histological appearance, with progressive changes to the glomerulus and tubulo-interstitium with increasing severity (Additional file 1). Milder disease (WHO classes II and IIIa) affects approximately 35% to 50%, whereas more serious classes IIIb, IV, and V affect 45% to 60% [7]. A significant minority of patients with class III disease (focal segmental proliferative glomerulonephritis) show worsening renal function and may progress to class IV lupus nephritis. Class IV (diffuse proliferative glomerulonephritis) usually presents with clinical evidence of renal disease, including oedema, hypertension, sediment, and worsening renal function with proteinuria. Class V (diffuse membranous glomerulonephritis) involves patients with laboratory and clinical features of nephrotic syndrome.

The aim of treatment is first to stop disease progression (induction phase) and then prevent recurrence (maintenance) while minimising the adverse effects. More specifically with lupus nephritis, the aims of treatment are to reduce the risk of end-stage renal disease, reduce renal and extra-renal lupus activity or symptoms, and reduce the mortality risk.

Treatment with immunosuppressive therapy is better than prednisolone monotherapy at preserving renal function, and combination therapy is better still [8-10]. Cyclophosphamide is used mainly to induce remission in patients with proliferative disease and has been considered to produce the best renal outcomes [11]. A systematic review of immunosuppressive treatments for lupus nephritis highlighted some of the problems [10]. These included ovarian failure, affecting 47% of women treated with cyclophosphamide plus steroid, and major infection in 20%. Nor were therapies entirely effective using cyclophosphamide plus steroid; doubled serum creatinine was observed in 24%, end-stage renal disease in 16%, and mortality in 21%. Weaknesses of these estimates included small numbers of trials and patients (maximum 290 in comparisons with steroid alone) and pooling data from patients with different disease severity who were being administered different regimens of immunosuppressive therapy. Few mentioned any blinding, but this is more difficult in long-term comparisons of parenteral and oral therapies.

Mycophenolate mofetil (MMF) is a relatively well-tolerated immunosuppressive agent used after kidney, liver, and heart transplants. MMF selectively and noncompetitively inhibits inosine monophosphate dehydrogenase in the *de novo *purine synthesis pathway. This enzyme facilitates the conversion of inosine monophosphate to xanthine monophosphate, an intermediate metabolite in the production of guanosine triphosphate. Because MMF results in the depletion of guanosine nucleotides, it impairs RNA, DNA, and protein synthesis [12]. Pharmacokinetics and metabolism of MMF suffer no major perturbations in renal impairment [13], but this may not be the case for severe renal insufficiency.

One year after renal transplantation, MMF at 2 or 3 g daily was at least as effective as azathioprine regimens at maintaining graft survival. Patient survival was similar, with a tendency to higher survival rates at longer duration [14,15]. MMF may be particularly beneficial in African-Americans [16]. MMF has also been shown to have some benefits over calcineurin inhibitors after liver transplantation [17]. MMF reduced mortality and graft loss compared with azathioprine over three years after heart transplantation in a large randomised trial [18]. Adverse events with MMF have tended to be different from those of other commonly used immunosuppressants. Diarrhoea may be more common [15,18] whereas ovarian failure is less common with MMF than with cyclophosphamide. The aim of this review was to systematically examine the available evidence concerning MMF in lupus nephritis from randomised trials and observational studies.

## Materials and methods

QUOROM (quality of reporting of meta-analyses) and MOOSE (meta-analysis of observational studies in epidemiology) guidelines were followed where applicable [19,20]. Studies of any design and in any language were sought concerning MMF in the treatment of patients with complications of lupus nephritis or SLE. A series of free text searches were undertaken to identify eligible reports from MEDLINE (to September 2005), Cochrane Library (Issue 3, 2005), and PubMed (to October 2006) by using the terms MMF or mycophenolate and the terms lupus nephritis or lupus erythematosus. Additional trials were sought in review articles [9,10,21-29] and reference lists of retrieved articles. The company (Aspreva Pharmaceuticals Ltd, Bagshot, Surrey, UK) developing CellCept for lupus nephritis was asked about the availability or knowledge of published or unpublished clinical trials. Authors of papers were not contacted for unpublished reports or additional information from published reports.

For completeness, we included studies in three separate groups: randomised trials, cohort studies with patients with SLE and lupus nephritis, and cohort studies with patients with SLE, only some of whom had lupus nephritis or undefined renal involvement. Full publications and abstracts were allowed. With multiple publications of the same study, we used the largest body of data or the most informative or the most recent (as appropriate). Excluded were reviews with clinical information published in a fuller form elsewhere, studies with purely biochemical (biomarker), pharmacokinetic, or immunological information, and studies in which MMF was used for treating other conditions.

Each randomised trial was scored for quality by using a three-item quality scale [30]. A maximum of five points were awarded to studies according to whether they were randomised, double-blind, and accounted for withdrawals or drop-outs and to whether the methods of randomisation and double-blinding were described and appropriate. No scoring system was used for cohort studies.

Two reviewers extracted information from the trials independently, and disagreement was resolved by consensus. Information extracted included the number of patients treated (per group in randomised trials), demographic information given (age, gender, and ethnicity), definition or classification of lupus nephritis (WHO was used), dosing regimens, duration of therapy, information about patients studied and their diagnoses, study design, the number of patients with efficacy and/or safety outcomes, and discontinuations, especially because of adverse events or lack of efficacy. Efficacy outcomes sought were those of complete or partial response to treatment as defined by the original authors (mainly urine protein excretion, serum creatinine or creatinine clearance, or a combination of these) and subsequent relapse. Adverse events sought included mortality, infection (especially severe infection), leucopaenia, gastrointestinal problems, amenorrhoea (in women), and hospital admission because of adverse events.

For analysis of efficacy, only those studies or trials with patients with both SLE and lupus nephritis were included. Any definition of lupus nephritis, as provided by authors, was accepted. If studies included patients who did not have lupus nephritis, efficacy information was only used for analysis if reported separately for nephritis patients. We used information on all patients for evaluation of adverse events because there was no prior indication that adverse events would be different in patients with SLE with or without nephritis or with different grades of nephritis.

For cohort studies, we calculated the percentage of patients with beneficial or harmful events by using as the denominator the total number of patients in studies mentioning the event. For randomised trials in which MMF was compared with another treatment, relative benefit and relative risk estimates were calculated with 95% confidence intervals by using a fixed effects model [31]. Because they have previously been shown to be unhelpful, heterogeneity tests were not used [32]; homogeneity was assessed visually [33]. Publication bias was not assessed using funnel plots, likewise because these tests have been shown to be unhelpful [34,35]. Relative benefit or risk was considered statistically significant when the 95% confidence interval did not include 1. The number needed to treat (NNT) and number needed to harm (NNH) with confidence intervals were calculated by the method of Cook and Sackett [36]. NNT or NNH values were calculated only when the relative risk or benefit was statistically significant.

The following terms were used to describe adverse outcomes with respect to harm or prevention of harm:

• The NNT to prevent one event (NNTp). We used this term when significantly fewer adverse events occurred with MMF than with the comparator.

• The NNH to cause one event. We used this term when significantly more adverse events occurred with MMF than with the comparator.

## Results

Additional file 2 has details of all the relevant studies found; there were no exclusions after screening, but not all studies were used in analyses. Six randomised trials in eight papers [37-44] reported on 370 patients in total, 197 of whom were treated with MMF. Ten cohort studies [45-54] reported on the use of MMF in 212 patients with lupus nephritis, and 8 cohort studies [55-62] reported on 284 patients with SLE treated with MMF, only some of whom had nephritis or renal problems. For several studies, there were multiple reports; duplicate information was avoided through detailed attention to authors, place of research, design, and study numbers. No study reported results separately for different classes of lupus nephritis or by ethnicity. Ethnicity was inconsistently reported (Additional file 2).

### Randomised trials

Two trials [37,38] were available as abstracts; one had [37] no useful data and was not included in any analysis. Two reports [41,42] refer to a 12-month trial using MMF followed by a switch to azathioprine [41], to which an extension with amended protocol with continued use of MMF was added [42]. It was not possible to separate results of the extension to the earlier trial, and for the purposes of data extraction, we used the later report, which included more patients [42].

The five studies used for analysis had quality scores of 2/5 (one trial) or 3/5 (four trials). They involved patients with SLE and lupus nephritis and used recognised diagnostic criteria for SLE and patients with biopsy-proven nephritis. Of the randomly assigned patients, 38 had WHO class III disease, 241 class IV, 27 class V, and 15 mixed membranoproliferative. Patients in the trials were predominantly (89%) women with average ages in the early or mid-30s. Information on ethnicity in four trials either was given or could be assumed from the origin of the study; of 306 patients, 27 were white, 106 black, 58 Hispanic, and 115 other, predominantly oriental.

Doses of MMF were in the range of 1 to 3 g daily, and doses were adjusted according to response and tolerability. Some trials reduced doses for maintenance therapy when remission was achieved, whereas in two [55,56], the mean daily doses were 2.7 and 1.6 g, respectively, without tailing. Corticosteroids were usually given together with MMF and comparators, usually with adjusted, tailing doses. Cyclophosphamide was the comparator in 199 of 218 patients and was given intravenously in 167 of 199 patients; azathioprine was used in 19 patients.

Two trials compared MMF and cyclophosphamide for induction treatment over the course of 6 to 9 months [43,44]. Two more [38,42] made the same comparison for induction followed by maintenance for up to 84 and 12 months, respectively, whereas a fifth [37] had tailing doses of MMF and appeared to involve both induction and maintenance. Only one trial [39] was clearly a maintenance regimen of MMF in a comparison with both cyclophosphamide and azathioprine, but with only 20 patients in each group.

### Cohorts

Eleven of the 18 cohort studies were available as full publications, and the other seven were available only as abstracts. Thirteen of the cohorts were prospective, four retrospective, and one [49] unclear. Patients in the 18 cohorts were predominantly (89%) women with age ranges generally from 16 to 60 years and average ages in the mid-30s. All described patients with SLE, predominantly fulfilling acknowledged diagnostic criteria, were inadequately controlled with corticosteroids, cytotoxics, cyclosporine, or antimalarial drugs.

Of 18 cohorts, 10 (212 patients [45-54]) included only individuals with lupus nephritis. Documentation of nephritis used biopsy and histology in five [50-54], but also deteriorating renal function, rising anti-double-stranded DNA titres, or inadequate control on conventional immunosuppressive therapy. Of the patients from the reports and descriptions, 14 had WHO class III disease, 136 class IV, and 62 class V. Information on ethnicity in eight cohorts either was given or could be assumed from the origin of the study; of 174 patients, 42 were white, 20 black, 1 Hispanic, and 111 other, predominantly oriental.

Eight other cohorts [55-62] included patients with SLE, some of whom had lupus nephritis; of 284 patients studied, 63 had WHO classifications for lupus nephritis (1 class II, 16 class III, 35 class IV, and 11 class V). Information on ethnicity in three cohorts either was given or could be assumed from the origin of the study; of 161 patients, 95 were white, 46 black, and 20 other.

Doses of MMF were between 125 mg and 3 g daily, but average or median doses were generally between 1 and 2 g daily. The duration of follow-up of patients on MMF was generally between 6 and 36 months, and average follow-ups were generally of 1 year or longer. None of the studies clearly indicated that they were specifically for induction or maintenance of renal remission. Corticosteroids were usually given together with MMF.

### Efficacy

#### Randomised trials

**Figure 1:**
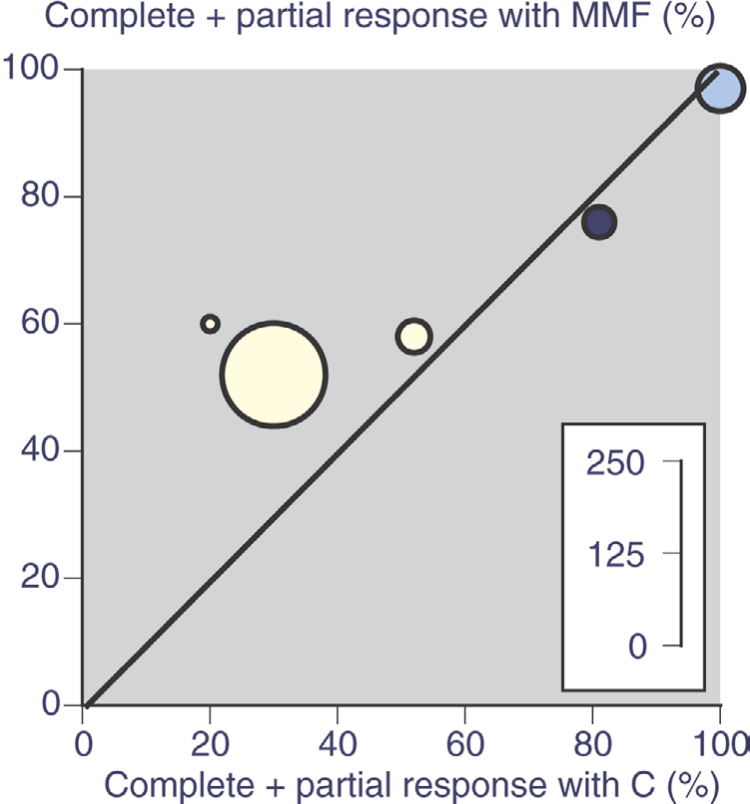
Complete plus partial response to oral mycophenolate mofetil (MMF) or intravenous or oral cyclophosphamide (C). The blue circles show trials with oral agents, with the sole maintenance trial using oral agents in dark blue. The inset scale represents the overall number of patients in each comparison.

Results for complete response and complete plus partial response in induction and maintenance therapy are shown in Table 1, in which the comparator was cyclophosphamide. There was a consistent response between studies. Figure 1 plots the proportion of patients in each trial with a complete and partial response with MMF or cyclophosphamide and shows the variability between individual trials.

MMF was significantly better than cyclophosphamide, with NNTs of approximately 8 for MMF compared with cyclophosphamide for one additional complete or complete plus partial response (that is, for every eight patients treated, one more achieved a response with MMF than with cyclophosphamide). Restricting the analysis to the four induction trials with 266 patients (omitting [41]) improved the NNT to 6.6 (95% confidence interval 3.7 to 30). The proportion of patients with complete or partial response in patients given MMF was 66%. Subsequent relapse after successful induction was reported in two trials, in 14/52 patients on MMF and 17/50 patients on cyclophosphamide.

#### Cohort studies

Complete or partial response to therapy with MMF was reported in 7 of the 10 cohort studies that recruited only patients with lupus nephritis. Complete or partial response was reported in 80% (Table 2) and failure or no response was reported in 20%. All 10 studies reported some measure of clinical or biochemical improvement. Reduced protein excretion was mentioned in 10, reduced corticosteroid dose in 7, with 3 each mentioning reduced serum creatinine and disease severity or increased serum albumin. Where numerical data were given, reduction in urinary protein excretion appeared to be of a major magnitude; average reductions were often greater than 50%, and reduction in average corticosteroid dose was also approximately 50% or more.

### Adverse events

#### Randomised trials

Death was reported less frequently with MMF (0.7%, 1 death in 152 patients) than with cyclophosphamide (7.8%, 12 deaths in 154 patients), with an NNTp of 14 (Table 1) (that is, for every 14 patients treated, 1 less death occurred with MMF than with cyclophosphamide).

Other adverse events occurred significantly less frequently with MMF than with cyclophosphamide, including all infections, serious infections, leucopaenia, amenorrhoea, and hair loss, with NNTp values of 3 to 10 compared with cyclophosphamide. Amenorrhoea was usually absent with MMF (Table 1). The information on all infections is dominated by the largest study [44], in which infection reporting was detailed but in which some patients may have had more than one infection. Diarrhoea occurred significantly more frequently (16%) with MMF than with cyclophosphamide (4%), with an NNH of 9. Adverse event discontinuations were not significantly different between the two treatments.

The number of days spent in hospital was 1 per patient per year with MMF or azathioprine in the maintenance trial [39] compared with 10 per patient per year for cyclophosphamide. Two induction studies [42,44] noted that the number of hospital admissions (usually after a serious adverse event such as vomiting and dehydration) was lower with MMF than with cyclophosphamide. There were 2 admissions for 115 patients with MMF compared with 16 admissions for 105 patients with cyclophosphamide. This rate was significantly lower for MMF compared with cyclophosphamide, with an NNTp of 7.

#### Cohort studies

Table 2 shows adverse event rates for all patients treated with MMF in all 18 cohort studies and for the set of 10 cohorts studies in which all patients had lupus nephritis and the set of eight additional studies in which only some had documented lupus nephritis or other renal problems. Adverse event rates were broadly similar in these two sets of cohorts, given the small number of events in some cases.

Overall, adverse event discontinuations averaged 14% and lack of efficacy discontinuations averaged 10% in the cohort studies (Table 1). Whereas any infection was common (23%), serious infections affected approximately 1 patient in 25, and approximately 1 in 30 had leucopaenia. Gastrointestinal symptoms of diarrhoea, nausea, and vomiting were common. Hair loss was reported in only two studies, with 5 cases in 42 patients. Amenorrhoea was not reported.

A single death occurred 1 year after a serious infection ([51]; Additional file 2). Assuming that death was an outcome likely to be reported in any study, this is a rate of 0.2% over the course of an average treatment period of at least 1 year.

## Discussion

Meta-analysis of small studies is problematic [63] because of possible inadequacies in design or reporting or because small numbers of patients and events increase the risk of chance findings [64]. The ideal is meta-analysis of high-quality, large, randomised trials [63], but without them we have to make the best of the information available and of all the information available. That is the justification, as here, for including information from both randomised trials and observational studies and (for adverse events) from observational studies in SLE in which only a proportion had lupus nephritis. Results, as here, can be reported separately to support any conclusions in which they are in concordance or to raise doubts about results in which they are not.

The background in lupus nephritis is one of few studies and small numbers. For cyclophosphamide and azathioprine, the mainstay of immunosuppressant therapy, there are at most 200 to 300 patients in randomised comparisons of immunosuppressant plus steroids with steroids alone [10]. In this analysis of MMF compared with cyclophosphamide for induction therapy, there were 197 patients treated with MMF in randomised trials. Ten cohort studies contributed information from an additional 212 patients.

Randomised trials, cohorts with lupus nephritis, and cohorts with SLE differed from each other (Table 3). Although the distribution of classes of lupus nephritis was broadly similar between randomised trials and cohorts with lupus nephritis, the ethic distribution was not. In randomised trials and cohort studies, whites were in a minority; in cohorts of SLE patients, they were in the majority. Even in randomised trials and cohorts with lupus nephritis, there were differences, with other race (principally oriental) predominating in cohorts with lupus nephritis.

**Figure 2:**
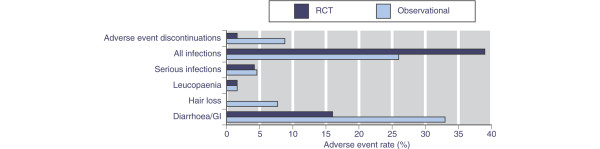
Comparison of adverse event rates with mycophenolate mofetil in systemic lupus erythematosus in a pooled analysis of randomised controlled trials (RCT) and observational studies reporting on patients with lupus nephritis. GI, gastrointestinal adverse events.

Despite these differences, the main findings of both randomised trials and cohort studies were much the same. Complete plus partial response with MMF occurred in 66% of patients in randomised trials and in 80% in cohorts, a similar proportion given the relatively small numbers. Mortality with MMF in randomised trials was of the same order of magnitude as in cohort studies (0.2%), with only two deaths. Adverse event rates were also similar (Figure 2), but numbers of events were small in many cases. For instance, hair loss in cohort studies depended on only five cases in two studies.

Adverse event rates in lupus nephritis patients were similar to those in patients with SLE (Table 2) and also to those found in renal [14,15] and heart [18] transplants. This provides additional reassurance of safety and tolerability of MMF, often at high doses (like the 3 g daily in the heart transplant trial) and over a treatment duration of up to 3 years. Despite this, there is the possibility of residual bias in observational studies and lack of blinding in randomised trials. The amount of information for MMF is greater than for cyclophosphamide and azathioprine in this indication, and trials, if anything, are somewhat better, but this is no defence against features of study design which we know may mislead [65].

Although the analysis provides information about efficacy and safety on average, it does not help determine which patients will respond best to MMF or other immunosuppressants. In most studies, either the requirement for entry was reasonable renal function (creatinine clearance greater than 30 ml/minute [44] or 20 ml/minute [39]) or renal function was relatively unimpaired (mean creatinine clearance was 80 ml/minute [43]). Patients with more severe renal disease were excluded, so we do not have information about efficacy or harm in the sickest patients, nor do we have much information about long-term maintenance rather than induction therapy.

Moreover, information was not reported separately for different racial groups or for different WHO classes of lupus nephritis, both of which are important considerations for determining appropriateness of therapy. Practitioners may be frustrated by the inability of a review to provide results for subgroups or better definitions of outcomes, but analysis can be performed only on average results unless individual results are available. The ideal would be an individual patient analysis based on collaboration between trialists.

Within these limitations, the evidence is that daily oral MMF is more effective than pulsed intravenous or oral cyclophosphamide. It produced more complete responses and complete plus partial responses, and the absolute difference, equivalent to an NNT of 8, was clinically useful. This better efficacy came with lower mortality, fewer hospital admissions, and lower rates of several adverse events, including severe infection. In a patient population of young women of childbearing age, it produced almost no cases of amenorrhoea and no cases of hair loss in randomised trials. Evidence from renal transplantation [66] is that MMF may also reduce cardiovascular risk relative to other immunosuppressants, and there are some theoretical reasons for this [67].

This is the first systematic review of MMF in lupus nephritis, but another is under way [68]. The previous systematic review by Flanc and colleagues [10] examined cyclophosphamide plus steroid versus steroid alone, often in older studies, and was limited, as here, by small numbers of events and patients. In that analysis, mortality with cyclophosphamide plus steroid was 21%, much higher than 1% or less with MMF, and approximately double the 7.8% mortality with cyclophosphamide plus steroid in the randomised trials in this review (Table 1). Serious infections with cyclophosphamide plus steroids occurred in 16% in the review of Flanc and colleagues (15% here), but amenorrhoea was higher (47%).

These results form a basis on which to plan future studies and provide a guide for the use of MMF in lupus nephritis until results of larger studies are available. Systematic reviews do not just provide an answer to a particular research question but should also highlight future research agendas. These would include reporting results not just for induction and maintenance, but for different classes of lupus nephritis and for populations with different risk factors such as ethnicity. At least one randomised trial is under way [69], with results from the induction phase expected in 2007, but it will be several years before the results of the maintenance phase are available.

## Conclusion

MMF produced more complete responses and complete plus partial responses, and the absolute difference was clinically useful. There are limitations to the existing data, not the least of which is to short-term results relative to the very long course of lupus nephritis.

## Abbreviations

MMF = mycophenolate mofetil; NNH = number needed to harm; NNT = number needed to treat; NNTp = number needed to treat to prevent one event; SLE = systemic lupus erythematosus; WHO = World Health Organization.

## Competing interests

RAM has received research funds, consultancy, and lecture fees from a number of pharmaceutical companies, charities, and government bodies. In the case of this study, work was supported by an unrestricted educational grant from Hayward Medical Communications (Newmark, UK) working on behalf of Aspreva Pharmaceuticals Ltd. Neither organisation had any role in design, planning, execution, or reporting of the study or the decision to publish it. The terms of the financial support included freedom for authors to reach their own conclusions and an absolute right to publish the results of their research, irrespective of any conclusions reached. Hayward Medical Ltd did have the right to view the final manuscript before publication and did so. Pain Research is supported in part by the Oxford Pain Research Trust.

## Authors' contributions

RAM was involved with the original concept, planning the study, data extraction, and analysis, and preparing a manuscript. SD was involved with data extraction, analysis, and writing. Both authors read and approved the final manuscript.

## Supplementary Material

Additional file 1 A PDF containing a brief summary of the WHO classification for lupus nephritis. Click here for file

Additional file 2 A PDF containing abstracted information about the cohort studies and randomised trials used in the review. Click here for file

## Figures and Tables

**Table 1:** Outcomes of randomised trials

Outcome	Number of trials	Number of patients	Percentage with MMF	Percentage with cyclophosphamide	Relative benefit or risk (95% CI)	NNT (95% CI)
Efficacy						
Complete response	4	266	36	23	1.5 (1.1 to 2.1)	7.6 (4.2 to 43)
Complete or partial response	5	306	66	54	1.2 (1.03 to 1.4)	8.0 (4.3 to 60)
Subsequent relapse	2	102	27	34	0.8 (0.4 to 1.4)	
Adverse events						NNTp (95% CI)
Death	5	306	0.7	7.8	0.2 (0.07 to 0.7)	14 (8 to 48)
Hospital admission	2	220	1.7	15	0.1 (0.04 to 0.5)	7.4 (4.8 to 16)
Adverse event discontinuations	3	246	1.6	5.6	0.3 (0.08 to 1.4)	
All infections	4	280	39	73	0.5 (0.4 to 0.7)	3.0 (2.3 to 4.4)
Serious infections	4	304	3.9	15	0.3 (0.1 to 0.6)	8.7 (5.5 to 21)
Leucopaenia	3	122	1.6	25	0.1 (0.03 to 0.5)	4.3 (2.9 to 8.3)
Amenorrhea	5	312	1.9	12	0.2 (0.08 to 0.6)	9.5 (6.2 to 20)
Hair loss	3	240	0.0	16	0.1 (0.01 to 0.4)	6.4 (4.4 to 11)
						NNH (95% CI)
Diarrhoea	4	260	16	4.0	4.0 (1.5 to 10)	8.5 (5.3 to 21)

CI, confidence interval; MMF, mycophenolate mofetil; NNH, number needed to harm; NNT, number needed to treat; NNTp, number needed to treat to prevent one event.

**Table 2:** Outcomes of cohort studies

	All cohort studies			Only definite nephritis			Any SLE patients		
Outcome	Number of cohorts	Number of events/patients	Percentage with outcome	Number of cohorts	Number of events/patients	Percentage with outcome	Number of cohorts	Number of events/patients	Percentage with outcome

Efficacy									
Complete or partial response	7	121/151	80	7	121/151	80	No data		
Failure/No response	8	37/176	21	7	30/151	20	1	7/25	28
Adverse events									
Adverse event discontinuations	9	40/285	14	4	6/68	8.8	5	34/217	16
Lack of efficacy discontinuations	5	24/235	10	1	4/24	17	4	20/211	9.5
All infections	10	76/327	23	4	18/70	26	6	58/257	23
Serious infections	8	8/185	4.3	4	4/87	4.6	4	4/98	4.1
Gastrointestinal (diarrhoea, nausea, vomiting)	14	122/406	30	8	52/160	33	6	58/257	29
Leucopaenia	8	6/188	3.2	4	1/61	1.6	4	5/127	3.9
Vertigo, dizziness	3	6/81	7.4	No data			3	6/81	7.4
Hair loss	2	5/42	12	1	1/13	7.7	1	4/29	14

SLE, systemic lupus erythematosus.

**Table 3:** Differences in patients recruited to different study types

				Percentage of ethnic origin			
Studies	Percentage with WHO class III	Percentage with WHO class IV	Percentage with WHO class V	White	Black	Hispanic	Other

Randomised trials	12	78	10	9	35	19	38
Cohort of lupus nephritis	7	64	29	24	11	1	63
Cohort of SLE	Not appropriate			59	29	0	12

SLE, systemic lupus erythematosus; WHO, World Health Organization.
