# Supplementary material for: Systematic review and meta-analysis of randomised trials and cohort studies of mycophenolate mofetil in lupus nephritis
Source: Arthritis Res Ther. 2006 Dec 12;8(6):R182. doi: 10.1186/ar2093 (PMC1794528; doi:10.1186/ar2093)
Supplement: Additional file 1 — A PDF containing a brief summary of the WHO classification for lupus nephritis. [file ar2093-S1.pdf]

**Additional file 1: WHO classification for lupus nephritis**

| Class | Designation | Comment |
|---|---|---|
| I | Normal | No evidence of lupus nephritis on the kidney biopsy. |
| II | Mesangial Nephritis | Most mild form of lupus nephritis; typically responds completely to treatment with corticosteroids. |
| III | Focal Proliferative Nephritis | Very early stage of more advanced lupus nephritis; typically treated with high doses of corticosteroids, with excellent outcome. |
| IV | Diffuse Proliferative Nephritis | Advanced stage of lupus nephritis with definite risk of loss of kidney function; typically treated with high doses of corticosteriods combined with immunosuppressive drugs. |
| V | Lupus Membranous Nephropathy | Generally associated with excessive protein loss and edema; typically treated with high doses of corticosteroids, with or without immunosuppressive drugs. |
