# Supplementary material for: Systematic review and meta-analysis of randomised trials and cohort studies of mycophenolate mofetil in lupus nephritis
Source: Arthritis Res Ther. 2006 Dec 12;8(6):R182. doi: 10.1186/ar2093 (PMC1794528; doi:10.1186/ar2093)
Supplement: Additional file 2 — A PDF containing abstracted information about the cohort studies and randomised trials used in the review. [file ar2093-S2.pdf]

Additional file 2: Details of cohort studies and randomised trials

Reference	Design, numbers, treatments, duration	Patients	Main efficacy outcomes	Main adverse event outcomes
**Randomised trials**				
Ye et al. 2001 Abstract 274<br><br>R=1<br>DB=0<br>WD=0	Randomised open comparison of:<br>MMF (45 patients) 1.5 g daily for 3 months, 1 g daily for 3 months, then 0.5 to 0.75 g daily<br>IV cyclophosphamide (45 patients)<br>0.75g/sq metre per month for 6-12 months<br>Prednisolone also available	Severe SLE with one or more of lung, renal, CNS involvement, hemolytic anaemia or vasculitic complications<br>No ethnicity given<br>WHO: no criteria given	MMF significantly improved clinical and laboratory parameters	GI intolerance, infection, leucopaenia, hair loss, liver dysfunction and ovarian failure less with MMF than cyclophosphamide
Flores-Suarez et al. ACR 2004 Abstract 1029<br><br>R=1<br>DB=0<br>WD=1	Randomised open comparison over 1 year of:<br>MMF (10 patients) up to 2 g daily<br>IV cyclophosphamide (10 patients) monthly<br>Prednisolone given to all and reduced according to response	Patients with lupus nephritis (type IV and V). Patients broadly similar initially<br>No ethnicity given<br>WHO: 17 IV, 3 V	Based on urine protein excretion and creatinine clearance, results at last follow up were:<br>Complete remission 1/10 IVC, 3/10 MMF<br>Partial remission 1/10 IVC, 3/10 MMF<br>Treatment failure 8/10 IVC, 4/10 MMF	Death: 3/10 IVC, 0/10 MMF<br>Leucopoenia: 5/10 , 1/10<br>Diarrhoea: 0/10, 2/10<br>Infections: 3/10, 5/10, (non severe)<br>Total alopecia: 1/10, 0/10<br>Amenorrhoea: 1/10, 0/10
Contreras et al. NEJM 2004 350: 971-980<br>Contreras et al. Lupus 2005 14: s33-s38<br><br>R=2<br>DB=0<br>WD=1	After IV cyclophosphamide induction, patients randomised to:<br>0.5-1 g/sq metre IV cyclophosphamide every three months (20 patients)<br>1-3 mg/kg azathioprine daily (19)<br>0.5 to 3 g MMF daily with dose titration (20)<br>Oral prednisone or other corticosteroid allowed<br>Duration 1-3 years<br>Median dose of MMF1.5 g/day during first 12 months, and then decreased	Patients with SLE according to ARA criteria, and with histological diagnosis of lupus nephritis after biopsy (WHO class III or greater)<br>No major differences between groups<br>Average age 32 years<br>55 women, 2 men<br>3W, 27B,30 H<br>WHO: 12 III, 46 IV, 1 V	Median duration of treatment was 25-30 months<br>Chronic renal failure<br>3/20 IVC, 1/19 AZ, 1/20 MMF<br>Renal relapse (increased protein creatinine ratio) 8/20 IVC, 6/19 AZ, 3/20 MMF<br>Event free survival better for AZ and MMF than IVC, and relapse-free survival better for MMF than IVC	Death: 4/20 IVC, 0/19 AZ, 1/20 MMF (mostly infections)<br>Hospital days per patient year 10 IVC, 1 AZ, 1 MMF<br>Adverse events per patient year back calculated to number beginning therapy:<br>Amenorrhoea: 6/18, 1/18, 1/19<br>Total infection: 15/20, 6/19, 6/20<br>Major infection: 5/20, 0/19, 0/20<br>Minor infection: 10/20, 5/19, 6/20<br>Leucopoenia: 2/20, 1/19, 0/20<br>Nausea: 13/20, 1/19, 3/20<br>Vomiting: 11/20, 1/19, 2/20<br>Diarrhoea: 2/20, 2/19, 2/20

Chan et al. NEJM 2000 343: 1156-1162.<br><br>R=1<br>DB=0<br>WD=1	After biopsy, patients randomised to:<br>oral cyclophosphamide 2.5 mg/kg/day plus prednisolone (21), replaced by azathioprine at 6 months, or<br>oral MMF 2 g daily plus oral prednisolone (21), with dose halved at 6 months, and replaced by azathioprine at 12 months	Patients with SLE according to ARA criteria, and with histological diagnosis of lupus nephritis after biopsy (WHO class IV)<br>No major differences between groups<br>Average age 37 years<br>39 women, 3 men<br>Presumed all Chinese<br>WHO: all IV	Based on urine protein excretion and creatinine clearance, results at 12 months were:<br>Complete remission 16/21 IVC/AZ,17/21 MMF<br>Partial remission 3/21 IVC/AZ, 3/21 MMF<br>Treatment failure 2/21 IVC/AZ, 1/21 MMF<br>Relapse 2/21 IVC/AZ, 3/21 MMF<br>There were major reductions in urinary protein excretion in both treatments, to average values of 0.5 and 0.2 g daily, and significant reductions in serum creatinine and increase in serum albumin	Death 2/21 IVC/AZ, 0/21 MMF<br>Infection 7/21 IVC/AZ, 4/21 MMF<br>Leucopoenia 2/21 IVC/AZ, 0/21 MMF<br>Hair loss 4/21 IVC/AZ, 0/21 MMF<br>Amenorrhoea 3/21 IVC/AZ, 0/21 MMF<br>Diarrhoea 0/21 IVC/AZ, 1/21 MMF<br>Adverse event discontinuations 1/21 IVC/AZ, 1/21 MMF<br>Hospital admissions for AE (episodes) 6/21 IVC/AZ, 3/21 MMF
Chan et al. J Am Soc Nephrol 2005 16:<br>[42 patients from Chan 2000 plus additional patients]<br><br>R=2<br>DB=0<br>WD=1	After biopsy, additional patients randomised to:<br>oral cyclophosphamide 2.5 mg/kg/day plus prednisolone (30), replaced by azathioprine at 6 months, or<br>oral MMF 2 g daily plus oral prednisolone (32), 1.5g daily at 6 months, and 1 g daily at 12 months<br>Maximum duration was 84 months	Patients with SLE according to ARA criteria, and with histological diagnosis of lupus nephritis after biopsy (WHO class IV)<br>No major differences between groups<br>Average age 39 years<br>52 women, 10 men<br>Presumed all Chinese<br>WHO: all IV	Response to induction was:<br>Complete remission 23/30 IVC/AZ, 24/32 MMF<br>Partial remission 7/30 IVC/AZ, 7/32 MMF<br>Longer term relapse: 9/30 IVC/AZ, 11/32 MMF, mostly with clear renal involvement.<br>Relapse-free survival the same in both maintenance groups:<br>any disease relapse 9/30 IVC/AZ, 11/32 MMF<br><br>End-stage renal disease 2/30 IVC/AZ, 0/32 MMF<br>Doubled creatinine 2/30 IVC/AZ, 2/32 MMF	Death: 2/30 IVC, 0/32 MMF<br>Severe infections:<br>1 in 103 patient months IVC/AZ, 1 in 234 patient-months MMF<br>Hospital admission for infection 1 in 177 patient months IVC/AZ, 1 in 328 patient-months MMF<br>Infections 12/30 IVC/AZ, 4/32 MMF<br>Infections needing hospital admission 9/30 IVC/AZ, 2/32 MMF<br>Composite end point of renal failure or death (2 deaths) 4/30 IVC/AZ, 0/32 MMF<br>Adverse event discontinuations 3/30 IVC/AZ, 1/32 MMF<br>Leucopoenia 8/30 IVC/AZ, 0/32 MMF<br>GI upset 1/30 IVC/AZ, 3/32 MMF<br>Severe hair loss 9/30 IVC/AZ, 0/32 MMF<br>Amenorrhoea 9/25 IVC/AZ (permanent in 5), 1/28 MMF
Ong et al. Nephrology 2005 10: 504-510.<br><br>R=2<br>DB=0<br>WD=1	Patients randomised to:<br>IV cyclophosphamide 0.75-1.0 g/sq metre for six months plus corticosteroids (25)<br>Oral MMF 2 g orally daily for six months plus corticosteroids (19)<br>Dose adjustements were allowed for adverse events, with dose reduction for prednisolone from 40-60 mg daily to maintenance of 5-10 mg daily<br>Mean daily dose of MMF was 1.6 g	Patients had newly diagnosed WHO III or IV lupus nephritis, and were aged 16 or older<br>Average age 31 years, and 37 of 44 patients were women<br>all oriental<br>WHO: 4 III, 40 IV	Remission in 13/25 IVC, 11/19 MMF<br>Complete remission 3/25 IVC, 5/19 MMF<br>Proteinuria decreased with both treatments, with upward trend in serum albumin.<br>Creatinine clearance rose over time, though was not different between treatments<br><br>Long-term follow up several years after the six-month induction period shwed no major differences between patients whose induction had been with IVC or MMF.<br>Patient survival was 94% and kidney survival 92% for both groups at 36 months	There were no deaths<br>Adverse event withdrawal 1/25 IVC, 0/19 MMF<br>End-stage renal failure 0/25IVC, 1/19 MMF<br>Oligomenorrhoea 1/25 IVC, 0/19 MMF<br>Serious infection 3/25 IVC, 3/19 MMF<br>Any adverse events 72 IVC, 68 MMF

Ginzler et al. NEJM 2005 353: 2219-2228<br><br>R=2<br>DB=0<br>WD=1	Patients randomised to:<br>IV cyclophosphamide 0.5 g/sq metre increasing to 1.0 g/sq metre, at monthly intervals (69)<br>Oral MMF 1 g daily to maximum of 3 g daily (71)<br>Duration was 3 months, and those with satisfactory response continued for a further 6 months, while those without a satisfactory response crossed to the other regimen, and after this period a further possible crossover was possible<br>All patients received prednisone or other corticosteroid<br>Mean daily dose of MMF 2.7 g daily	Patients had ACR criteria for SLE, and biopsy-proven class IV-VI lupus nephritis with evidence of active nephritis<br>Average age 32 years<br>126 women, 14 men<br>24W, 79B, 28H, 9 other<br>WHO: 22 III, 76 IV, 27 V	Response to induction was:<br>Complete remission 4/69 IVC, 16/71 MMF<br>Partial remission 17/69 IVC, 21/71 MMF<br>No remission at 24 weeks on initial regimen 21/69 IVC, 19/71 MMF<br>Serum creatinine and urine protein excretion fell with both treatments, and serum albumin increased. Oral corticosteroids could be tapered over 3-6 months<br><br>After about 36 months follow up, renal failure or death were twice as frequent after IVC (7 and 8 cases respectively) than after MMF (4 cases each)	Death: 2/69 IVC, 0/71 MMF<br>Withdrawal treatment failure 3/69 IVC, 5/71 MMF<br>Total withdrawal 24/69 IVC, 15/71 MMF<br>Adverse event withdrawal 3/69 IVC, 1/71 MMF<br><br>At least one adverse event 24/75 IVC, 20/83 MMF (including crossover)<br>Severe infection 6/75 IVC, 1/83 MMF<br>Any infection 68/75 IVC, 42/83 MMF<br>Upper GI symptoms 25/75 IVC, 23/83 MMF<br>Diarrhoea 2/75 IVC, 15/83 MMF<br>Amenorrhoea 2/75 IVC, 0/83 MMF<br>Alopecia 8/75 IVC, 0/83 IVC<br>Sustained lymphopenia 14/75 IVC, 5/83 MMF<br>Nausea and vomiting hospital admission 7/75 IVC, 0/83 MMF<br>Rectal bleeding 3/75 IVC, 0/83 MMF
**Cohort studies in SLE patients with only lupus nephritis**				
Dooley et al. J AM Soc nephrol 1999 10: 833-839.	Prospective cohort of 13 patients followed up for 2-24 months<br>MMF doses began at 0.5 to 2 g daily, to maximum of 1.0 to 2.5 g daily	Resistant or relapsing lupus nephritis, after previous therapy<br>Age range 16-48 years<br>9 women, 4 men<br>8W, 4B, 1 H<br>WHO: 12 IV, 1 V	Over 13 months average treatment, there was a significant reduction in proteinuria (PC ratio fell from 5.5 to 2.9), and serum creatinine was stable or improved (mean fall 149 to 123 μmol/L)<br>Abnormal urine sediment in 13/13 initially, 7/13 at 13 months<br>Prednisone dose reduced in 10/13, eliminated in 2/13	1/13 discontinued because of pancreatitis (MMF-related)<br>1/13 had period of leucopaenia<br>1/13 had scalp hair loss<br>3/13 mild nausea or diarrhoea<br>1/13 severe nausea, vomiting and diarrhoea
Kingdon et al. Lupus 2001 10: 606-611.	Prospective cohort of 13 patients followed up for 6-37 months<br>Median maintenance dose was 1 g daily (range 250 mg to 3 g daily)	All satisfied ACR criteria for SLE, and nine had deteriorating renal function. All had previously relapsed on conventional treatment, or to minimise steroid dose and avoid further cytotoxic use<br>Age range 20 to 47 years<br>All women<br>4W, 3B, 6 other<br>WHO: 1III, 6 IV, 6 V	Over 25 months average treatment, nephrotic relapse occurred in 1/13 , though MMF was re-introduced<br>Disease severity scores fell in 12/13<br>Mean proteinuria fell from 1.5 g daily to 0.9 g daily<br>Oral corticosteroids reduced 6/10 or stopped 2/10	1/13 withdrew non-compliance<br>1/13 withdrew because of severe nausea<br>3/13 moderate/severe infections<br>2/13 GI symptoms<br>0/13 severe leucopaenia or anaemia
Hu et al. Chin Med J 2002 115: 705-709.	Prospective open non-randomised trial in 46 patients followed for 45 months<br>MMF initial dose 1-1.5 g daily, reduced to 0.5 to 1 g daily<br>Cyclophosphamide intravenously followed by pulsed therapy to dose of 2-3 grams, followed by prednisone 0.8 mg/kg/day. IV cyclophosphamide at 0.75 to 1.0 g/sq metre given monthly for six months, then quarterly	All had SLE according to ARA criteria, urine sediments and proteinuria, renal biopsy showing lupus nephritis, and without severe concomitant disease<br>Average age 29 years<br>38 women, 8 men<br>Presumed all Chinese<br>WHO: all IV	Over 45 months follow up, 21/23 on MMF had more than 50% reduction in urinary RBC, and 16/21 in urinary protein excretion. For 8/23, urine protein excretion was below 0.5 g daily<br>Repeat renal biopsy showed improvements	Adverse events were:<br>2/23 herpes zoster<br>6/23 GI symptoms<br>4/23 infections<br>0/23 leucopaenia

Spetie et al. Kidney Int 2004 66: 2411-2415.	Prospective cohort of 13 patients with nephritis followed up for 4-30 months<br>MMF doses were 1 g daily initially, then 1.5 to 2 g daily by 2-4 weeks	SLE patients satisfied at least four ACR criteria, with no immunosuppressive therapy before onset of nephritis<br>Age range 20-60 years<br>11 women, 2 men<br>7W, 5B, 1 other<br>WHO: all V	Mean treatment duration was 16 months. By six months of therapy, 10/13 achieved complete (8) or partial (2) remission, with 11/13 at longest follow up.<br>Oral prednisone reduced in 12/13 patients<br>Reduction in proteinuria	Serious adverse event 1/13 infection<br>1/13 mild diarrhoea<br>0/12 leucopaenia or anaemia
Kim et al. EULAR 2004, Abstract 0360	Cohort of 46 patients with lupus nephritis treated with MMF for at least 3 months<br>Dose not given	No additional patient details given<br>All Korean<br>WHO: implied IV/V	Complete remission 26/46<br>Partial remission 7/46<br>Relapse after complete remission 2/46<br>No response 13/46<br>Improved ESR and serum albumin, decreased proteinuria and steroid dose reported	Gastrointestinal problems affected 28/46, with hair loss, leucopaenia, anaemia, infection, myalgia and headache mentioned, but no severe adverse events
Ding et al. Lupus 2004 13: 113-118.	Prospective cohort of 9 patients with lupus nephritis followed up for at least 6 months<br>MMF dose 1-2 g daily for three months, with subsequent reductions	Renal biopsy confirmed lupus nephritis<br>Age range 14-39 years<br>6 women, 3 men<br>Presumed all Chinese<br>WHO: all IV	After 6 months, all 9 patients were free of systemic symptoms, with significant fall in protein excretion and haematuria. Disease activity indices improved<br>4/9 complete remission (protein excretion <1 g daily)<br>4/9 partial remission (protein excretion 1-2 g daily)<br>1/9 failed treatment	Gastrointestinal adverse events were described as tolerable
Kapitsinou et al. Rheumatol 2004 43: 377-380.	Retrospective cohort of 18 patients with lupus nephritis followed up for up to 31 months<br>MMF dose 2 g daily	All patients had SLE according to ACR criteria, with biopsy-proven lupus nephritis. 12 patients had proliferative lupus nephritis and 6 lupus membranous nephropathy<br>Average age 32 years<br>17 women, 1 man<br>No ethnicity given<br>WHO: 9 III, 6 IV, 3 V	After an average of 15 months of treatment:<br>10/18 complete remission (protein excretion <0.5 g daily)<br>4/18 partial remission (protein excretion <2 g daily, or 30% or more reduction)<br>4/18 failures (all membranous nephropathy)<br>Significant reduction in urine protein excretion, sediment, and serum creatinine, with increased creatinine clearance<br>Steroid dose reduced in 14/18 patients, from 15mg daily initially to 7 mg at end of follow up	1/18 developed a serious infection, and died 1 year later<br>2/18 gastrointestinal intolerance needing dose changes
Borba et al. ACR 2005 Abstract 1129	Prospective cohort of 20 patients with SLE and proteinuria followed up for an average of 12 months<br>Initial MMF dose of 1.5 g daily increased to 2-3 g daily after one month	Main inclusion criterion refractory proteinuria despite corticosteroids. 12 patients had biopsy documented membranous glomerulonephritis<br>Average age 35 years<br>No ethnicity given<br>WHO: all V	Partial response (≥50% decrease in proteinuria) in 20/20, with mean protein excretion down from 3.5 g to 1.3 g daily, and significant increase in serum albumin, after 8 months<br>Complete response (normal urine protein excretion of <0.3 g daily) in 11/20 after 12 months, with mean urine protein of 0.2 g daily.<br>No response in 3/20 patients<br>Oral prednisone fell from an average of 34 mg to 2 mg daily	No information given

Karim et al. Rheumatology 2005 44: 1317-1321.	Retrospective cohort of 10 patients, with follow up 3-52 months<br>MMF starting dose 0.5 g daily to maximum of 1.0 to 2.5 g daily	Biopsy-proven lupus membranous nephropathy<br>9/10 fulfilled ACR criteria for SLE<br>Previous therapy azathioprine, cyclosporine, and cyclophosphamide<br>Age range 30-49 years<br>8 women, 2 men<br>5W, 5B<br>WHO: all V	Mean treatment duration 19 months<br>Disease severity scores fell in 3/6 patients<br>ESR fell in 7/8 patients<br>Serum creatinine fell in 6/10<br>Urine protein excretion fell in 9/10 patients, especially at higher initial excretion rate (median 2.3 initially to 0.7 at last follow up<br>Serum albumin levels increased significantly<br>Daily prednisolone reduced in 8/10	2/10 infections (not obviously severe)<br>5/10 GI symptoms<br>1/10 discontinuation due to GI symptoms
Cross et al. Nephron Clin Pract 2005 100:c92-c100.	Prospective cohort of induction regimen in 24 patients with lupus nephritis for 1 year (19 completed 1 year)<br>MMF initially 1 g daily, to 2 g daily over 4 weeks, with prednisolone	All patients had renal biopsy proven lupus nephritis. No patient previously treated with MMF or cyclophosphamide<br>Average age 33 years<br>21 women, 3 men<br>18W, 3B, 3 other<br>WHO: 4 III, 11 IV, 9 V	At 6 months, 20/24 had complete renal remission , 2/24 partial remission, 2/24 refractory<br>At 12 months 5/20 now had only partial remission<br>Urine protein excretion fell from 3.9 to 1.3 g daily, while serum albumin rose from 28 to 37 g/L. Anti-dsDNA titres also fell.	5/24 withdrawn (2 refractory, 1 serious infection, 1 flare in extra-renal disease, 1 continuing disease).<br>Other adverse events were:<br>9/24 infections<br>4/24 GI<br>3/24 venous thrombosis<br>1/24 thrombocytopoenia

**Cohort studies that included SLE patients without lupus nephritis**				
Gaubitz et al. Lupus 1999 8: 731-736.	Prospective cohort of 10 patients with follow up 8-16 months<br>MMF 1.5 or 2 g daily, depending on weight	ACR defined SLE, >12 SLAM, 16-65 years<br>Corticosteroids, antimalarials, and other therapies used at start. 4/10 with nephritis<br>Age range 16-46 years<br>All women<br>No ethnicity given<br>WHO: 1 II, 3 IV	Over 11 months average treatment, SLAM 16 initially reduced to 10 and 8 at 3 and 6 months; 8/10 >20% reduction in SLAM at 6 months<br>SLE-related erythema improved in 5/5, and arthritis in 5/6<br>ESR reduced, and mean prednisolone dose reduced from 10 to 5 mg daily	No significant change in blood markers<br>No adverse event discontinuations<br>4/10 diarrhoea initially<br>2/10 prurigo<br>2/10 vertigo<br>0/10 severe infections
Karim et al. Rheumatol 2002 41: 876-882.	Prospective cohort of 21 patients followed up for 0.5 to 33 months<br>MMF starting dose was 0.5 g daily to a maximum of 2 g daily	At least four of ACR criteria for SLE, mostly previously treated with immunosuppressants. 13 patients had active renal disease at entry<br>Age range 21 to 47 years<br>20 women, 1 man<br>12W, 8B, 1 other<br>WHO: 1II, 4 III, 6 IV, 2 V	Over 14 months average treatment, disease activity scores fell from 13 initially to 4 at the last visit<br>Proteinuria fell from 3.7 to 1.1 g daily<br>Prednisolone reduced from mean 20 mg daily to 10 mg daily (15/16)	3/21 withdrew because adverse events, mainly gastrointestinal<br>2/21 wished to become pregnant<br>2/21 moderate or severe infections<br>1/21 leucopaenia
Doria et al. ACR abstract 1464, 2003	Prospective cohort of 42 patients, followed up for 2-24 months (28 followed up for 9 months)<br>MMF dose was 2 g daily with prednisone	SLE patients with:<br>glomerulonephritis (15)<br>thrombocytopoenia (2)<br>arthritis (6)<br>CNS involvement (2)<br>glomerulonephritis after standard induction therapy (14)<br>Age range 21-49 years<br>37 women, 5 men<br>No ethnicity given<br>WHO: all IV	Over 11 months average treatment, there was a significant improvement in disease activity scores and reduction in prednisone dose. Proteinuria was reduced in patients with glomerulonephritis after induction therapy	Adverse events were:<br>6/42 adverse event withdrawals<br>3/42 treatment failure withdrawals<br>5/42 infections<br>4/42 nausea<br>3/42 abdominal pain<br>2/42 diarrhoea<br>3/42 dizziness, headache<br>1/42 leucopaenia
Riskella et al. J Rheumatol 2003 30: 1508-1512.	Retrospective consecutive patients (54), followed up for an average of 12 months<br>MMF dose ranged from 125 mg to 3 g daily	SLE patients with confirmed diagnosis receiving MMF, about two-thirds receiving azathioprine or cyclophosphamide at some time. 26/54 with active renal disease at start of MMF therapy<br>Average age 37 years<br>50 women, 4 men<br>39W, 14B, 1 other<br>WHO: no criteria given	Over 12 months average treatment there were significant improvements on disease activity scores, and significant reduction in daily prednisone (from 20 mg to 12 mg daily) with stable creatinine	Any adverse event 36/54, probably related to MMF<br>2/54 serious adverse events<br>1/54 serious infection<br>21/54 gastrointestinal<br>24/54 infections<br>3/54 leucopaenia<br>Withdrawals:<br>9/54 adverse event<br>3/54 lack of efficacy<br>2/54 pregnancy<br>2/54 other

Shekshina et al. EULAR 2003 abstract no.	Prospective cohort of 36 patients, with 29 completing at least 6 months treatment<br>MMF 2 g daily	SLE patients inadequately controlled with corticosteroids, cytotoxics, cyclosporine or antimalarials; 19 patients had lupus nephritis<br>Age range 17-51 years<br>32 women, 4 men<br>No ethnicity given<br>WHO: no criteria given	Overall improvements for clinical disease scoring, white cell counts, and serum protein and albumin in those with lupus nephritis. Proteinuria improved in 6/15 with nephritis.<br>Also improved were erythema (3/9), skin vasculitis (2/4), arthritis/arthralgia (4/11), headache (5/12)<br>Oral prednisolone decreased from mean 18 to 15 mg daily	Adverse events were:<br>2/29 treatment failure<br>4/29 adverse events (dizziness, nausea, allergy, thrombocytopoenia)<br>4/29 hair loss<br>2/29 nausea<br>2/29 abdominal pain<br>2/29 infections
Bijl et al. Ann Rheum Dis 2003 62: 534-539.	Prospective cohort of 10 patients with SLE with rising anti-dsDNA antibodies treated for 6 months with MMF<br>MMF dose 2 g daily	SLE patients satisfied at least four ACR criteria<br>Age range 22-63 years<br>9 women, 1 man<br>No ethnicity given<br>WHO: no criteria given	No patient had a clinical relapse on MMF. Significant fall in anti-dsDNA antibodies over treatment period. Patient assessment of improvement significant from 2-6 months	Minor hypertensive event in one patient requiring increased therapy, and one unconfirmed fever<br>0/10 leucopaenia or anaemia
Frassi et al. EULAR 2005 Abstract 0455	Prospective cohort of 25 patients with renal and nonrenal lupus resistant to conventional immunosuppressive therapy for 3-42 months<br>MMF dose 0.5-2 g daily	All previously treated with various immunosuppressive drugs<br>Age range 21-54 years<br>24 women, 1 man<br>No ethnicity given<br>WHO: no criteria given	After a median treatment duration of 18 months patients had reduced disease activity. Proteinuria and creatinine clearance not significantly different. No effect in 7/25 patients<br>Significant reduction in oral prednisone from 12 to 11 mg daily	Adverse events in 14/25 patients<br>5/25 discontinued because of adverse events<br>7/25 GI disorders<br>5/25 infections<br>1/25 CNS lymphoma<br>1/25 hypoglycaemia
Pisoni et al. J Rheumatol 2005 32: 1047-1052.	Retrospective cohort of 86 patients with SLE, 59 with lupus nephritis followed up for an average of 16 months<br>Maximum MMF dose was 0.5 to 2.5 g daily	All patients had SLE according to ACR criteria. 59 had lupus nephritis and 29 uncontrolled disease activity. All patients had at least one immunosuppressive drug before MMF<br>Average age 37 years<br>76 women, 10 men<br>44W,24B,18 other<br>WHO: 11 III, 12 IV, 9 V	In all patients, there were reductions in steroid dose and activity index, and improvement in ESR. In 35 renal patients there was reduced proteinuria from 3.0 to 1.9 g daily.	Discontinuations because of adverse events occurred in 14/86 patients, mostly because of GI complaints or infections<br>12/86 discontinued because of lack of efficacy,<br>6/86 pregnancy<br>Other adverse events were:<br>37/86 at least one adverse event<br>25/86 GI symptoms<br>20/86 infections<br>2/86 depression<br>1/86 haematological
